# Self-Reported Frailty Concerns in Older Breast Cancer Survivors During the COVID-19 Pandemic

**DOI:** 10.1093/geroni/igab046.3437

**Published:** 2021-12-17

**Authors:** Adele Crouch, Diane Von Ah

**Affiliations:** 1 University of Pennsylvania, Philadelphia, Pennsylvania, United States; 2 Indiana University, Indianapolis, Indiana, United States

## Abstract

Frailty among older adults is common, especially those who have undergone breast cancer treatment; however, we do not know how frailty among this group presented during the COVID-19 pandemic. The purpose of this descriptive, cross-sectional study was to examine self-reported frailty among older breast cancer survivors (BCS) during the pandemic. This IRB-approved study recruited BCS who were at least 1-year post-treatment and 60 years of age or older, via online advertisements (e.g., Dr. Susan Love Foundation). BCS completed demographic and Tilburg Frailty Indicator (TFI) RedCap questionnaires from 11/2020 to 05/2021. The TFI, is a 15-item measure with 3 sub-scales with published cut points indicating frailty: total (5), physical (3), psychological (2), and social (2). Descriptive statistics were used. Older BCS (n=203) who were on average 65.5 (SD=4.7) years of age, white (93.6%; n=190) and had stage II breast cancer at diagnosis (39.9%; n=81) participated. The average total (M=5.4, SD=2.5) and physical (M=3.2, SD=1.5) frailty scores were above the threshold for frailty. Overall, 58.6% (n=119) and 63.1% (n=128) scored at or above the threshold on the total and physical sub-scales, respectively. In addition,78.8% (n=160) responded that they ‘missed having people around’ on the social frailty sub-scale. Research has shown that higher TFI scores (more frailty) are associated with increased healthcare utilization, poorer quality of life, and even mortality. Thus, frailty among older BCS is an important health concern within the context of the pandemic. Further research is needed to understand the lasting effects of self-reported frailty for BCS including COVID-19 survivors.

